# The Influence of Peripheral Neuropathy, Gender, and Obesity on the Postural Stability of Patients with Type 2 Diabetes Mellitus

**DOI:** 10.1155/2014/787202

**Published:** 2014-09-02

**Authors:** Aline Herrera-Rangel, Catalina Aranda-Moreno, Teresa Mantilla-Ochoa, Lylia Zainos-Saucedo, Kathrine Jáuregui-Renaud

**Affiliations:** ^1^Unidad de Investigación Médica en Otoneurología, Instituto Mexicano del Seguro Social, Planta Baja del Edificio C-Salud en el Trabajo del Centro Médico Nacional Siglo XXI, Avenida Cuauhtémoc 330, 06720 Colonia Doctores, DF, Mexico; ^2^Hospital Regional 72, Instituto Mexicano del Seguro Social, Avenida Gustavo Baz Esquina Filiberto Gomez, 54000 Tlalnepantla, MEX, Mexico; ^3^Hospital de Pediatría, Instituto Mexicano del Seguro Social, Centro Médico Nacional Siglo XXI, Avenida Cuauhtémoc 330, 06720 Colonia Doctores, DF, Mexico

## Abstract

*Aim.* To assess the influence of peripheral neuropathy, gender, and obesity on the postural stability of patients with type 2 diabetes mellitus.* Methods.* 151 patients with no history of otology, neurology, or orthopaedic or balance disorders accepted to participate in the study. After a clinical interview and neuropathy assessment, postural stability was evaluated by static posturography (eyes open/closed on hard/soft surface) and the “Up & Go” test.* Results.* During static posturography, on hard surface, the length of sway was related to peripheral neuropathy, gender, age, and obesity; on soft surface, the length of sway was related to peripheral neuropathy, gender, and age, the influence of neuropathy was larger in males than in females, and closing the eyes increased further the difference between genders. The mean time to perform the “Up & Go” test was 11.6 ± 2.2 sec, with influence of peripheral neuropathy, gender, and age.* Conclusion.* In order to preserve the control of static upright posture during conditions with deficient sensory input, male patients with type 2 diabetes mellitus with no history of balance disorders may be more vulnerable than females, and obesity may decrease the static postural control in both males and females.

## 1. Introduction

Intact balance is required to maintain postural stability as well as to assure safe mobility during activities of daily life. Balance corrections imply the interaction among several sensory inputs and the major contributor during quiet upright stance may be somatosensory inputs [1, 2]; information from the legs is utilized for both direct sensory feedback and use of prior experience in scaling the magnitude of automatic postural responses [3].

A frequent cause of peripheral neuropathy is type 2 diabetes mellitus [4]. In this group of patients, the frequency of balance symptoms may be related to both the time elapsed since the diabetes was diagnosed and the history of peripheral neuropathy and retinopathy [5]. Assessment of postural control during upright stance has shown that patients with diabetes and peripheral neuropathy may sway more than those without peripheral neuropathy [6–8]. In addition, men may exhibit more spontaneous sway than women [9] and adults with obesity may have a decrease in postural stability with a larger dependency on vision to control balance [10]. Increased body mass may produce instability [11]; subjects with a body mass index greater than 30 maintain shorter times in balance and longer times unbalanced as compared with lean individuals [12]. Even further, in subjects with obesity, weight loss seems to improve measures of static postural stability [13].

The aim of this study was to assess the influence of peripheral neuropathy, gender, and obesity on the postural stability of patients with type 2 diabetes mellitus, receiving primary health care.

## 2. Patients and Methods

After the study was approved by the institutional research and ethics committee, 151 consecutive patients with type 2 diabetes mellitus receiving primary health care gave their informed consent to participate. None of them were seeking medical care due to balance decline or had history of otology, neurology, psychiatry, or orthopaedic or balance disorders. All of them denied receiving ototoxic medication. Patients were aged 38 to 80 years (mean 57.1 ± S.D. 9.4 years), 107 were females (57 ± 8.8 years old), and 44 were males (57.5 ± 11.1 years old); 48% (95% C.I. 38.1–57.9%) of them had systemic high blood pressure. The mean age when diabetes was diagnosed was 47.7 ± 10 years and the time elapsed since diabetes was diagnosed was 9.3 ± 5.7 years. Their mean glucose serum level was 146.7 ± 55.2 mg/100 mL, and 64.8% (95% C.I. 57.2–72.4%) of them had HbA1c >7%; the most frequent medication was metformin (86%, 95% C.I. 80.5–91.5%) and 22% (95% C.I. 15.4–28.6%) of the patients required insulin.

The mean body mass index (BMI) of the patients was 29 ± 4.8. However, 92 patients had a BMI <30 (59 ± 9.2 years old), 42 patients had a BMI from 30 to <35 (55.4 ± 9.4 years old), and 17 had a BMI ≥35 (51.6 ± 8.1 years old).

Peripheral neuropathy was evaluated at first by the Michigan Diabetic Neuropathy Score [14] and the Semmes-Weinstein 10 g monofilament; when any of these two instruments were positive, nerve conduction studies were performed (Spirit, Nicolet, Madison) [15].

Postural stability was evaluated by body sway during static posturography and the timed “Up & Go” test [16]. Body sway during quiet upright stance was recorded at 40 Hz using a force platform (Posturolab 40/16 Medicapteurs, Cedex); each trial lasted for 51.2 sec and, during this period, subjects were asked to stand upright and barefoot on the platform as still as possible with arms at their sides. Recordings were made under 4 conditions, while adding or not a layer of foam rubber (5 cm thick, density of 2.5 pcf) to the base of support, with the eyes either open or closed [17]: condition 1 = hard surface and eyes open; condition 2 = hard surface and eyes closed; condition 3 = soft surface and eyes open; condition 4 = soft surface and eyes closed. Before each trial, the feet were positioned according to the manufacturer reference, and small adjustments were made online; recordings with the eyes closed were obtained just after acquiring the data with the eyes open, without moving the feet. To perform the “Up & Go” test, patients were asked to stand from a chair with armrests, walk 3 meters, turn, and go back to their seat at their normal pace. A standard digital stopwatch was used to record the time to the nearest tenth of a second, from the command to “go” to the time when the backsides of the patient touched the chair.

Statistical analysis was performed using *t*-test and analysis of covariance. The significance level was set at 0.05. To perform the analysis of covariance, the BMI of the patients was classified as follows: <30, 30 to <35, and ≥35; the age and the number of males/females according to the BMI group are described in Table 1.

## 3. Results

Peripheral neuropathy was diagnosed in 32 patients (21.2%, 95% C.I. 14.7–27.7) who had a similar age compared to patients without peripheral neuropathy (56.7 ± 10.5 years old versus 57.2 ± 9.2) but had a longer time of evolution of the disease (11.8 ± 6.2 years versus 8.6 ± 5.5 years) (*t*-test; *P* = 0.005). A motor component was evident only in 3 patients. The percentage of patients with peripheral neuropathy according to the BMI group is described in Table 1, which was more frequent in those with a BMI ≥30, but with no significant difference among the subgroups.

**Figure 1:**
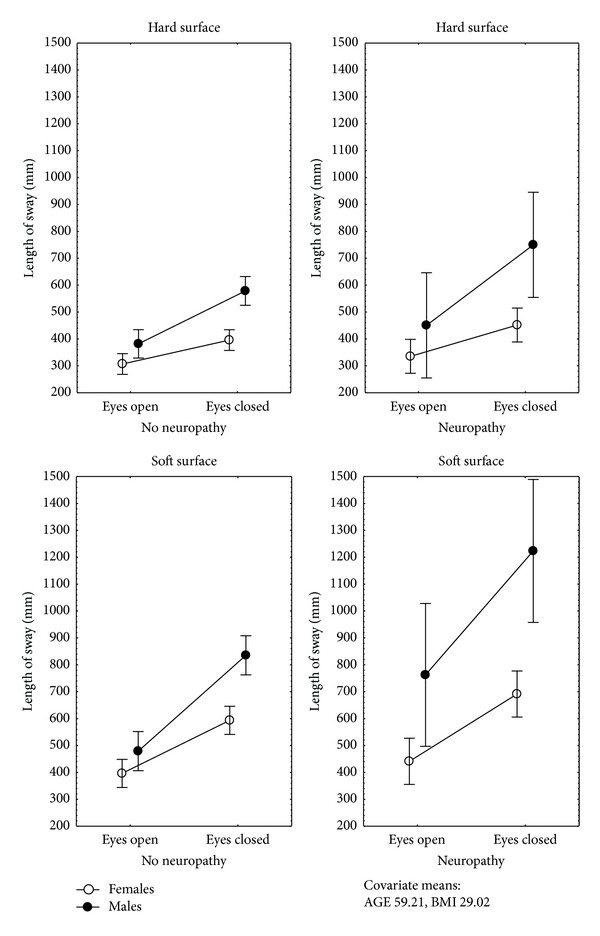
Mean and standard error of the mean of the length of sway during static posturography, by gender, evidence of neuropathy, and sensory condition of 151 patients with type 2 diabetes mellitus receiving primary health care.

The characteristics of sway are described in Table 2. Since the area of sway showed high variability among groups and conditions, significant results were observed only in the length of sway (MANCoVA, *P* < 0.05), with an influence of the following variables:

- condition 1 (multiple *R* = 0.5; *P* < 0.001): the gender (beta = 0.41, 95% C.I. 0.05–0.77) (Figure 1) and the age of the patients (beta = 0.36, 95% C.I. 0.21–0.52), with no significant interactions;
- condition 2 (multiple *R* = 0.44; *P* = 0.001): the gender (beta = 0.66, 95% C.I. 0.29–1.03) (Figure 1), the BMI group (beta = 0.58, 95% C.I. 0.14–1.01), and the evidence of peripheral neuropathy (beta = 0.42, 95% C.I. 0.08–0.77); an interaction between the BMI group and the evidence of peripheral neuropathy was observed (beta = 0.58, 95% C.I. 0.11–1.06);
- condition 3 (multiple *R* = 0.52, *P* < 0.01): the gender (beta = 0.54, 95% C.I. 0.19–0.89) (Figure 1), the evidence of peripheral neuropathy (beta = 0.35, 95% C.I. 0.02–0.68), and the age (beta = 0.35, 95% C.I. 0.19–0.50), with no significant interactions;
- condition 4 (multiple *R* = 0.47; *P* < 0.001): the gender (beta = 0.74, 95% C.I. 0.38–1.11) (Figure 1), the evidence of peripheral neuropathy (beta = 0.46, 95% C.I. 0.12–0.80), and the age (beta = 0.2, 95% C.I. 0.05–0.36); an interaction between the gender and the evidence of peripheral neuropathy was observed (beta = 0.40, 95% C.I. 0.01–0.79).

These results were consistent when comparing the recordings with the eyes open or closed, either on hard or on soft surfaces as follows.

**Figure 2:**
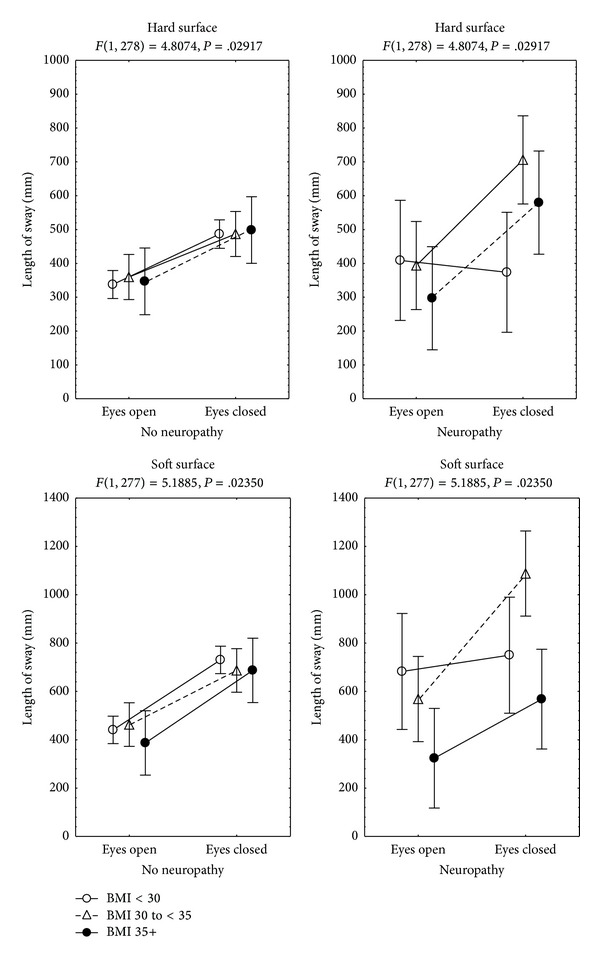
Mean and standard error of the mean of the length of sway during static posturography, by body mass index group, evidence of neuropathy, and sensory condition of 151 patients with type 2 diabetes mellitus receiving primary health care.

- *Hard Surface*. There was an influence of (multiple *R* = 0.53; *P* < 0.001) the gender (beta = 0.51, 95% C.I. 0.27–0.76), the BMI group (beta = 0.38, 95% C.I. 0.09–0.67), the evidence of peripheral neuropathy (beta = 0.3, 95% C.I. 0.07–0.59), and the age (beta = 0.17, 95% C.I. 0.06–0.28); an interaction between the BMI group and the evidence of peripheral neuropathy was observed (beta = 0.34, 95% C.I. 0.2–0.65) (Figure 2).
- *Soft Surface*. There was an influence of (multiple *R* = 0.62; *P* < 0.001) the gender (beta = 0.58, 95% C.I. 0.35–0.8), the evidence of peripheral neuropathy (beta = 0.36, 95% C.I. 0.15–0.57), and the age (beta = 0.12, 95% C.I. 0.12–0.32); an interaction between the gender and the evidence of peripheral neuropathy was observed (beta = 0.32, 95% C.I. 0.08–0.56).

The mean time to perform the “Up & Go” test was 11.6 ± 2.2 sec. Analysis of covariance showed that the time to perform the test had an influence of (multiple *R* = 0.3; *P* < 0.001) peripheral neuropathy (beta = 0.23, 95% C.I. 0.018–0.045), the gender (beta = 0.16, 95% C.I. 0.018–0.32), and the age (beta = 0.17, 95% C.I. 0.01–0.33).

## 4. Discussion

The results of this study show that, in patients with type 2 diabetes mellitus, during upright stance, the influence of peripheral neuropathy and vision on the length of sway may be more evident in male than in female patients, particularly while standing on a soft surface, and obesity may have a further influence on sway in the two genders, while standing on a hard surface, when vision is not available. However, when performing a standardized daily-life task “Up & Go test”, the influence from obesity may not be evident, even when peripheral neuropathy and the gender have an influence.

To maintain stability when moving from one sensory context to another, it is important to reweigh the sensory information depending on the context. In healthy subjects, increased severity of experimentally induced loss of plantar cutaneous sensitivity may be associated with greater postural sway; such an association could be affected by the availability of visual input and the size of the support surface [18]. In this study, we observed that, in patients with type 2 diabetes mellitus, the influence of peripheral neuropathy on the length of sway is related to the gender and to obesity, with larger sway in male than in female patients and a larger increase of sway after closing the eyes in obese subjects than in nonobese subjects.

In the present study, in patients with peripheral neuropathy, static posturography showed that closing the eyes while standing on hard surface had a larger effect in patients with a BMI ≥30 than in those with a BMI <30, and while standing on a soft surface, patients with a BMI ≥35 had less sway than patients with a BMI <35. In contrast, an independent influence of neuropathy was observed during all the sensory conditions of the study. Although peripheral neuropathy was more frequent among patients with a BMI ≥30, it was similar among those with a BMI from 30 to <35 and those with a BMI ≥35 (Table 1). In subjects without diabetes mellitus, evidence has shown that, during quiet standing, obese subjects have an increase of the peak pressure on fore-foot and plantar ground contact area [19], and compared to control and overweight subjects obesity may be related to a decrease in postural stability, when vision is not available, suggesting that obese subjects may be more dependent on vision to control balance [10]. Additionally, evidence has shown that, after closing the eyes, the increase of sway in obese subjects may be similar when recordings are made either on hard or on soft surface [10], suggesting that obese subjects may use their somatosensation to control posture differently than lean and overweight subjects, which is consistent with the results of the present study.

Although the influence of age was evident during all the sensory conditions and the “Up & Go” test, it is already known as well that sway increases with increasing age [20, 21], with an increased dependence on vision [22, 23]; the results of this study suggest that the influence of peripheral neuropathy, obesity, and gender on the length of sway may be not dependent on age. Since male patients had a similar age compared to female patients, patients with peripheral neuropathy, as well, had a similar age compared to patients without neuropathy and patients with obesity were even younger than nonobese patients (Table 1).

The finding of a larger sway in males than in females is consistent with previous reports showing that men may exhibit more spontaneous sway than women, and this difference may increase when there is no visual input [9, 10]. In addition, in this study we observed that the difference between genders may be increased by peripheral neuropathy and by distortion of somatosensory inputs (soft surface conditions).

The findings of this study should be interpreted in the context of its limitations. Since the study has a cross-sectional design, imprecision of any real association may be possible. The enrolment was limited to patients requiring primary health care, so the results may not apply to patients with more physical impairments.

## 5. Conclusion

In order to preserve the control of static upright posture during conditions with deficient sensory input, male patients with type 2 diabetes mellitus with no history of balance disorders may be more vulnerable than females and, in both males and females, obesity may decrease their static postural control.

## Figures and Tables

**Table 1:** Mean and standard deviation of the mean of the age of 151 patients with type 2 diabetes mellitus, with the number of males/females and the frequency of peripheral neuropathy, according to the BMI group.

Variables	BMI < 30	BMI 30 to < 35	BMI ≥ 35
	(*n* = 92)	(*n* = 42)	(*n* = 17)
Age (mean ± S.D.)	59 ± 9.2	55.4 ± 9.4	51.6 ± 8.1
Males/females	26/66	12/30	6/11
Peripheral neuropathy (%)	14.1%	33.3%	29.4%

**Table 2:** Mean and standard deviation of the mean of the sway characteristics of 151 patients with type 2 diabetes mellitus during upright stance on hard/soft surface, with the eyes open/closed.

Variables	BMI < 30 (*n* = 92)		BMI 30 to < 35 (*n* = 42)		BMI ≥ 35 (*n* = 17)	
	Eyes open	Eyes closed	Eyes open	Eyes closed	Eyes open	Eyes closed
	Mean ± S.D.	Mean ± S.D.	Mean ± S.D.	Mean ± S.D.	Mean ± S.D.	Mean ± S.D.
Hard surface						
Length (mm)	330 ± 104	448 ± 219	347 ± 126	481 ± 292	332 ± 99	522 ± 243
Area (mm^2^)	105 ± 99	215 ± 671	108 ± 82	198 ± 295	139 ± 112	280 ± 267
*X* ^a^ position (mm)	4.5 ± 36.8	0.75 ± 6.3	0.09 ± 6.2	0.69 ± 6.2	0.32 ± 4.8	0.67 ± 4.7
*Y* ^b^ position (mm)	−29.6 ± 19.1	−28.2 ± 15.5	−34.6 ± 19.5	−32.4 ± 18.5	−36.8 ± 14.2	−33.1 ± 12.1
Soft surface						
Length (mm)	447 ± 189	686 ± 308	452 ± 197	724 ± 385	368 ± 91	652 ± 252
Area (mm^2^)	204 ± 283	413 ± 505	216 ± 210	505 ± 521	234 ± 229	420 ± 361
*X* ^a^ position (mm)	−0.01 ± 7.2	−0.68 ± 7.6	2.12 ± 6.4	1.06 ± 6.4	−1.59 ± 4.3	−1.82 ± 5.7
*Y* ^b^ position (mm)	−26.6 ± 17.5	−25.3 ± 15.9	−33.2 ± 20.9	−29.5 ± 24.5	−32.9 ± 14	−31.2 ± 13.9

^a^ *X*: lateral-lateral position of the centre of pressure.

^b^ *Y*: anterior-posterior position of the centre of pressure.
